# SELDI-TOF MS profiling of serum for detection of nasopharyngeal carcinoma

**DOI:** 10.1186/1756-9966-28-85

**Published:** 2009-06-17

**Authors:** Yuan-Jiao Huang, Chao Xuan, Bei-Bei Zhang, Ming Liao, Kai-Feng Deng, Min He, Jin-Min Zhao

**Affiliations:** 1 Guangxi Medical Scientific Research Center, Guangxi Medical University, Nanning, PR China; 2 Department of Orthopedics Trauma and Hand Surgery, the First Affiliated Hospital, Guangxi Medical University, Nanning, PR China

## Abstract

**Background:**

No satisfactory biomarkers are currently available to screen for nasopharyngeal carcinoma (NPC). We have developed and evaluated surface-enhanced laser desorption/ionization time-of-flight mass spectrometry (SELDI-TOF MS) for detection and analysis of multiple proteins for distinguishing individuals with NPC from control individuals.

**Methods:**

A preliminary learning set and a classification tree of spectra derived from 24 patients with NPC and a group of 24 noncancer controls were used to develop a proteomic model that discriminated cancer from noncancer effectively. Then, the validity of the classification tree was challenged with a blind test set, which included another 20 patients with NPC and 12 noncancer controls.

**Results:**

A panel of 3 biomarkers ranging m/z 3–20 k was selected to establish Decision Tree model by BPS with sensitivity of 91.66% and specificity of 95.83%. The ability to detect NPC patients was evaluated, a sensitivity of 95.0% and specificity of 83.33% were validated in blind testing set.

**Conclusion:**

This high-flux proteomic classification system will provide a highly accurate and innovative approach for the detection/diagnosis of NPC.

## Background

Nasopharyngeal carcinoma (NPC) is a disease that has remarkable racial and geographic distribution [1]. It is rare in Europe and North America. However, it has a high incidence in several southern areas in China, especially in the provinces of Guangdong, Guangxi, Hunan and Hong Kong Special Administrative Region et al [2]. The phenomenon indicates that the development of this cancer must be related to special genetic and environmental factors.

NPC is highly sensitive to radiotherapy (RT) and chemotherapy (CT), but the outcome is related to the extent of the disease. Unfortunately, most patients with NPC are diagnosed at stage III or IV NPC when they visit the otorhinolaryngologists. Therefore, early detection and diagnosis of NPC is crucial for a better outcome of the patients [3].

Routine clinical methods of examination for nasopharyngeal diseases, such as the use of nasoendoscopy, are not applicable as a screening tool because can be used only by an otorhinolaryngologist and are not cost effective. Epstein-Barr virus (EBV) infection is consistently associated with NPC, and is classified as a group I carcinogen by the International Agency for Research on Cancer (IARC) [4,5]. Serological tests, detecting the antibodies to Epstein-Barr virus (EBV), such as viral capsid antigen (VCA) immunoglobulin A (IgA), early antigen (EA) IgA, and Epstein-Barr nuclear antigen (EBNA1) IgA have been used routinely as serological screening markers in high-risk populations. Nevertheless, none of them has proven to be a stand-alone and reliable assay due to either low sensitivity or specificity [6,7]. Therefore, identification of additional biomarkers is important for the early detection and management of this disease.

The proteome reflect all proteins and peptides that may be related with one gene and allows a more detailed evaluation of disease status using the human proteome. At present, it has become relatively easy to detect the protein profiling in the crude biological samples with surface-enhanced laser desorption/ionization-time of flight mass spectrometry (SELDI-TOF MS). The proteomic technique was first introduced by Hutchens and Yip in 1993 [8], and applied to protein chips with different chromatographic affinities in serum. This is a high-throughput technical plateform which can detect multiple protein changes simultaneously with high sensitivity and specificity [9,10].

In the present study, by comparative analysis of patients with NPC and noncancer controls, using Ciphergen SELDI Software 3.1.1 with Biomarker Wizard, some potential serum NPC-associated proteins biomarkers were discovered, which might be new candidate biomarkers for NPC diagnosis. At the same time, the diagnostic model was established which could effectively differentiate NPC patients from noncancer controls.

## Methods

### Study population

The serum samples of 80 patients collected between October 2007 and April 2008 were provided by First Affiliated Hospital, Guangxi Medical University. The only selection criterion for patients was that their NPC diagnosis had been confirmed pathologically. The diagnosis of all patients was poorly differentiated squamous cell carcinoma. The control group comprised 36 noncancer normal volunteers who visited the General Health Check-up Division at First Affiliated Hospital, Guangxi Medical University. Selection criteria for controls were no evidence of any personal or family history of cancer or other serious illness. All NPC patients and noncancer donors involved in the study signed an agreement form consenting to the donation of their specimens. The demographics of the NPC patients and controls were shown in Table 1. From each sample, 8 ml blood was allowed to clot at 4°C for at least 2 h and then centrifuged at 1500 g for 10 min to sediment the clotted cells. Serum was collected, divided into aliquots, and stored frozen at -80°C until ProteinChip array profiling analysis was carried out.

**Table 1:** Demographics of The Study Population

Groups	stage	Number	Sex(M/F)	MedianAge (Years)
NPC	I&II	16	9/7	49
	III & IV	28	18/10	53
Noncancer Normal		36	15/21	45

NPC indicates nasopharyngeal carcinoma

### Serum Protein Profiling

Each serum sample was analyzed on four different ProteinChip arrays surfaces: hydrophobic (H50), cation-exchange (CM10), anion-exchange (Q10) and metal binding (IMAC30-Cu). In addition, sinapinic acid (SPA) was used as energy absorbing molecule (EAM) on all surfaces in parallel experiments. The CM10 chip was found to attain the highest number of protein peaks among the chips tested. Therefore, it was suitable for this work and used throughout the study. Serum samples were thawed and briefly centrifuged (5 minutes, 10,000 revolutions per minute [rpm]) and pretreated before loading. To 10 μl of each serum sample, 20 μl U9(5 μl of a solution containing 8 mol/L urea and 10 g/L CHAPS in 1×phosphate-buffered saline(PBS) [pH 7.2])was added. The mixture was incubated with vigorous shaking at 4°C for 30 minutes. After incubation, the diluted serum mixture was mixed with 360 μl binding/washing buffer (0.1 M sodium acetate, [pH 4.0]). Place the ProteinChip array cassette in the bioprocessor and add 200 μl binding solution to each well. Incubate for 5 minutes at room temperature with vigorous shaking (e.g., 250 rpm or on Micromix shaker setting 20/7), Repeat once. Remove the buffer from the wells. Immediately add 100 μl sample to each well. Incubate with vigorous shaking for 1 hour at room temperature. Remove the samples from the wells, and wash each well with 200 μl binding buffer for 5 minutes, with agitation. Repeat once. Remove the binding buffer from the wells, and add 200 μl HEPES (50 mM hydroxyethyl piperazine ethanesulfonic acid, [pH4.0]) to each well; remove immediately. Then, the ProteinChip was removed from the bioprocessor and dried at room temperature. Apply 1 μl of SPA (sinapinic acid [Sigma Chemical, St. Louis, MO] in 50% acetonitrile volume/volume (v/v) and 0.5% v/v trifluoroacetic acid) Energy Absorbing Molecules (EAM) in solution to each spot. Air-dry for 5 minutes and apply another 1 μl of SPA in solution. Allow to air-dry.

### SELDI-TOF MS Analysis

Mass/charge (m/z) spectra of proteins with affinity to the Weak Cation Exchanger surface were generated in a Ciphergen Protein Biology System (PBS-IIc) plus TOF-MS Reader (Ciphergen Biosystems). Data were collected by averaging the results of a total of 200 laser shots with an intensity of 180, a detector sensitivity of 8, a high mass to m/z 100 k and an optimization range of m/z 2–20 k. Mass curacy was calibrated externally using the All-in-One peptide mass standard (Ciphergen Biosystems) and SELDI-TOF-MS analysis was performed on the same day.

### Data Analysis

The entire dataset was randomly separated into training and test datasets before analysis. A training set consisted of spectra data from 24 patients with NPC and 24 noncancer controls to build up the classification tree. The discriminatory ability of the classification algorithm was then challenged with a blind test dataset consisting of another spectra data of another 32 serum samples. All spectral data were normalized by total ion current after background subtraction. The range of peak masses was analyzed between m/z 2–20 k because the majority of resolved protein/peptides were found in this range. The molecular masses from m/z 0–2 k were excluded from analysis because they were mainly the signal noises of the energy absorbing molecule (EAM). The Biomarker Wizard (Ciphergen Biosystems) was subsequently used to make peak detection and clustering across all spectra in the training set with the following settings: signal/noise (first pass): 5; minimum peak threshold: 15% of all; mass error: 0.3%; and signal/noise (second pass): 2 for the m/z 2–20 k mass range. Corresponding peaks in the spectra from the test set were likewise identified using the clustering data from the training set by the same software. The spectral data of the training set were then exported as spreadsheet files and then further analyzed by the Biomarker Pattern Software (BPS) (version 4.0; Ciphergen Biosystems) to develop a classification tree.

### Decision Tree Classification

One of the objectives of SELDI-TOF MS data analysis is to build a Decision Tree that is able to determine the target condition (case or control, cancer or non-cancer) for a given patient's profile. Peak mass and intensity were exported to an excel file, then transferred to BPS. The classification model was built up with BPS. A Decision Tree was set up to divide the training dataset into either the cancer group or the control group through multiple rounds of decision-making. When the dataset was first transferred to BPS, the dataset formed a "root node". The software tried to find the best peak to separate this dataset into two "child nodes" based on peak intensity. To achieve this, the software would identify the best peak and set a peak intensity threshold. If the peak intensity of a blind sample was lower than or equal to the threshold, this peak would go to the left-side child node. Otherwise, the peak would go to the right-side child node. The process would go on for each child node until a blind sample entered a terminal node, either labeled as cancer or control. Peaks selected by the process to form the model were the ones that yielded the least classification error when these peaks were combined to be used. The double-blind sample dataset was used to challenge the model. Peaks from the blind dataset were selected with Biomarker Wizard feature of the Software, following the exact conditions under which peaks from the training dataset were selected. The peak intensities were then transferred to BPS, and each sample was identified as either control or cancer based on the model. The results were compared to clinical data for model evaluation. To characterize the protein peaks of potential interest, serum profiling of patients with NPC and normal control was compared. Mean peak intensity of each protein was calculated and compared (nonparametric test) in each group of serum samples [11].

### Statistical analysis

Sensitivity was calculated as the ratio of the number of correctly classified diseased samples to the total number of diseased samples. Specificity was calculated as the ratio of the number of negative samples correctly classified to the total number of true negative samples.

## Results

### Reproducibility and precision

To assess the precision and accuracy of the proteomic data in our analyses, we employed external calibration standards using all-in-one peptide molecular mass standard (Ciphergen Biosystems, Inc. Ciphergen Biosystems, Inc. USA), allowing us to achieve a mass accuracy of approximately 0.1%. To confirm the reproducibility of our analyses, we compared 10 selected M/Z peaks from an unaffected person. The coefficient of variance for the selected M/Z peaks with the highest amplitude was less than 15%.

### Serum SELDI profiles of NPC versus nocancer normal controls

**Figure 1:**
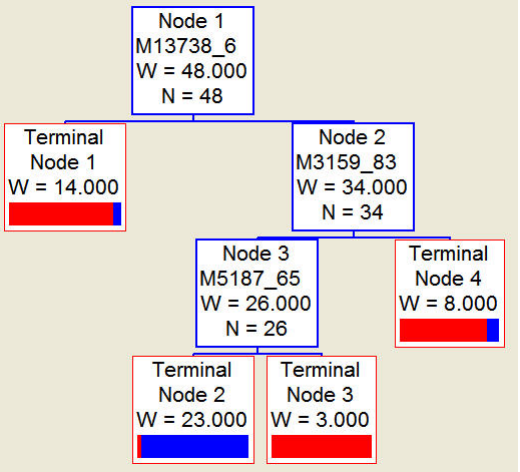
**Diagram of a decision tree for the classification of the nasopharyngeal carcinoma (NPC) and noncancer samples in the learning dataset**. The circles indicated the primary nodes and the squares were the terminal nodes. The mass value in the root nodes was followed by the intensity value.

**Figure 2:**
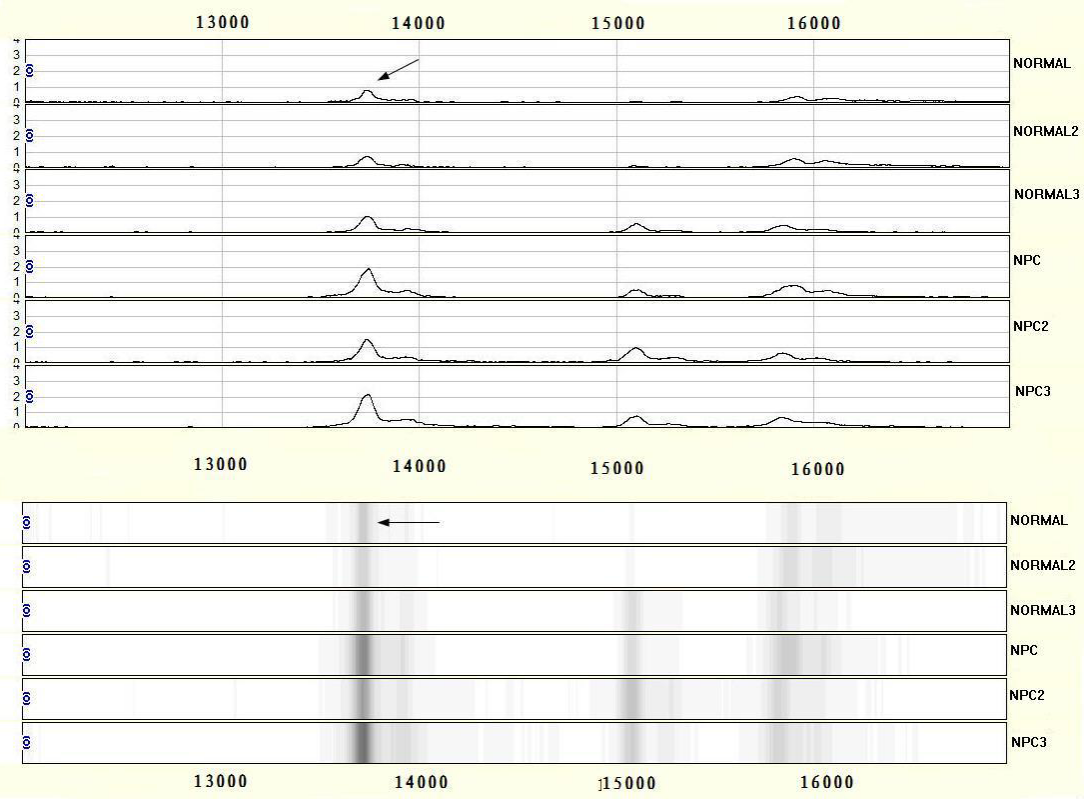
**SELDI analysis of serum for proteomic pattern in samples from patients with nasopharyngeal carcinoma (NPC) and in control samples with mass spectra and gel view**. The x-axis represents the molecular mass calculation (mass-to-change ratio [m/z]) and the y-axis represents the relative intensity. The mass spectrographic profiles reveal up-regulation of the m/z 13738.6.

**Figure 3:**
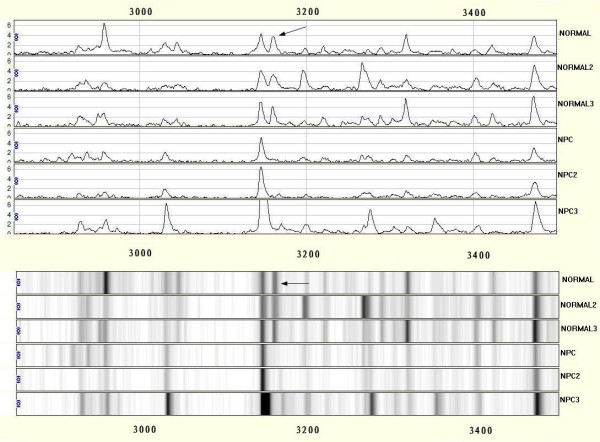
**SELDI analysis of serum for proteomic pattern in samples from patients with nasopharyngeal carcinoma (NPC) and in control samples with mass spectra and gel view**. The x-axis represents the molecular mass calculation (mass-to-change ratio [m/z]) and the y-axis represents the relative intensity. The mass spectrographic profiles reveal peaks in NPC samples and down-regulation of the m/z 3159.835.

**Figure 4:**
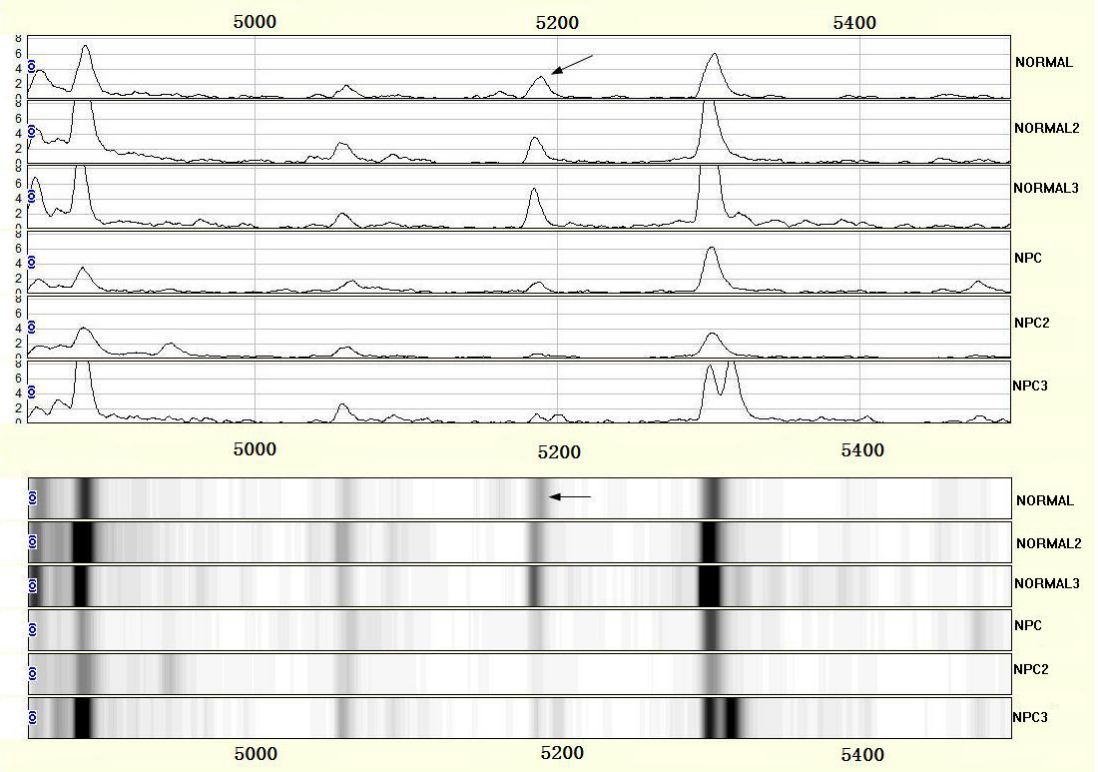
**SELDI analysis of serum for proteomic pattern in samples from patients with nasopharyngeal carcinoma (NPC) and in control samples with mass spectra and gel view**. The x-axis represents the molecular mass calculation (mass-to-change ratio [m/z]) and the y-axis represents the relative intensity. The mass spectrographic profiles reveal m/z 5187.656 peaks.

**Figure 5:**
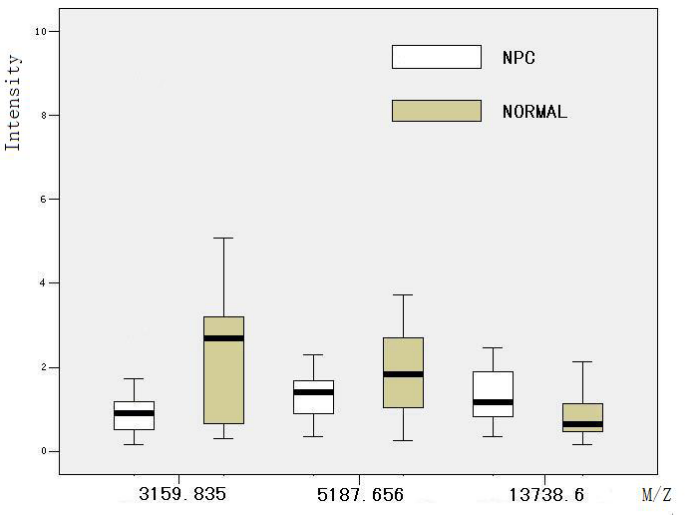
**Relative peak intensities of m/z 3159.835, 5187.656, 13738.6 protein masses in serum samples from patients with nasopharyngeal carcinoma (NPC) compared with samples from the noncancer controls**. Results are shown as box-and-whisker plots.

After noise filtering and peak cluster identification, 94 mass peaks were detected in the training set. These peak data from the training set were saved and exported for pattern recognition by the BPS. The most optimal Decision Tree with the highest accuracy eventually was established. The Decision Tree used 3 splitters with distinct masses of m/z 3159.83 5187.65 13738.6 respectively, and classified the cases into 3 lymph nodes (Figure. 1). The peaks at m/z 13738.6 were highly expressed in NPC but weakly expressed in healthy individuals, and the other 2 peaks were highly expressed in healthy individuals but weakly expressed in patients with NPC. The descriptive statistics of these 3 Biomarkers were shown in Table 2. Characteristic spectrum graphs of 3 Biomarker were shown in Figure 2, Figure 3, and Figure 4 and the descriptive statistics are shown in Figure 5.

**Table 2:** Statistical Analysis of 3 Biomarkers for Screening Patients With Nasopharyngeal Carcinoma Versus Healthy Controls

	Intensity, mean ± SD		
		
Protein peaks, m/z	Noncancer normal	NPC	*P*
3159.835	2.13 ± 1.44	1.22 ± 1.04	0.017728
5187.656*	2.00 ± 1.31	1.38 ± 0.60	0.094881
13738.6	0.86 ± 0.54	1.31 ± 0.60	0.002791

SD indicates standard deviation; m/z, mass-to-change ratio; NPC, nasopharyngeal carcinoma.

*The peak is necessary for Decision Tree although the *P *value > 0.05.

The error rate of the generated Decision Tree was estimated through a process of cross-validation. Performance of the generated Decision Tree is summarized in Table 3 for the training and test sets. A blind test set, which consisted of samples from 20 patients with cancer and 12 noncancer controls, was used to evaluate the ability of Diagnostic Pattern to distinguish between patients with NPC and noncancer controls. In our study, 10 of 12 true noncancer control samples were classified correctly, and 19 of 20 cancer samples were classified correctly as malignant. This set result yielded a sensitivity of 95%, a specificity of 83.33%, and an accuracy rate of 90.63% (Table 3).

**Table 3:** Performance of the Decision Tree Analysis of NPC in Training Test and Blind test Sets

	Sensitivity,%	Specificity, %	Accuracy rate, %
Training set	91.66(22/24)	95.83(23/24)	93.75(45/48)
Test set	87.5(21/24)	95.83(23/24)	91.67(44/48)
Blind test set	95.0(19/20)	83.33(10/12)	90.63(29/32)

## Discussion

Currently, there are no satisfactory serum diagnostic markers for NPC, especially in the early stage [12]. Complex serum proteomic patterns might reflect the potential pathological state of a disease such as NPC and enable the scientific community to develop more reliable diagnostic tools. In this study, we used SELDI-TOF MS technology to disclose the serum protein 'fingerprints' of NPC and thereby establish a new diagnostic model for NPC.

SELDI-TOF MS allows the identification of large numbers of potential biomarkers in a biological sample, based on molecular weights and chemical characteristics. In essence it provides high throughput screening for biomarkers, particularly when present in low abundance, avoiding the limitations of antibody binding and of only analyzing predetermined proteins. It is able, therefore, to identify proteins not previously appreciated to be potentially valuable biomarkers. The technology has been applied to serum and urine to identify disease specific biomarkers [13]. However, the number of peaks that can be identified by this approach does not cover the whole serum proteome. This is related to several potential technical limitations. A more complete proteome could be obtained by depleting serum of the most abundant proteins, preliminary fractionation of serum before analysis by SELDI-TOF MS, or by testing several different ProteinChip arrays. However, these technical limitations are counterbalanced by the high efficiency and ease of use of the system, which makes SELDI-TOF MS a useful tool for clinical proteomics [14].

The tumorigenesis of NPC is a complex, multistep process that involves multiple genetic mutations [15]. In light of the multifactorial nature of NPC, it is plausible that a combination of multiple biomarkers will be necessary to improve the diagnosis of NPC. Our study has identified 94 potential biomarkers and established a protein diagnostic pattern to distinguish NPC from noncancer controls with a specificity of 95.83% and a sensitivity of 91.66%. The accuracy rate of this pattern was 93.75%. Among the 3 biomarkers, the m/z with m/z 3159.835 5187.656 were down-regulated in the cancer group, and the m/z with 13738.6 was up-regulated in the cancer group. In the blind test, the sensitivity was 95.0% and the specificity was 83.33%. These results suggest that this pattern of biomarkers can be used for the early detection and screening of NPC. Further research is needed to identify the 3 unknown m/z protein species in the serum profiles of our patients and to confirm our current findings in larger cohorts of study samples.

All together, the SELDI-TOF MS ProteinChip technology can demonstrate that biomarkers are present in patients with NPC and help establish differential patterns with high sensitivity and specificity. Done reproducibly in multiple laboratories and the analysis is amenable to simultaneous analysis of dozens or hundreds of samples. In addition to the current work detailed here, similar results have been demonstrated in another recent publication [15-17] and techniques to further improve data quality for robust peak identification have also been described [18]. These features establish SELDI analysis as a powerful approach to proteomic analysis in population based studies, and hence the utility of this technology can be exploited in all phases of the NPC studies.

## Competing interests

The authors declare that they have no competing interests.

## Authors' contributions

JMZ: corresponding author, study design. YJH: literature search, Screen four different ProteinChip arrays, data analysis of mass-spectrum, revise the article. CX: literature search, serum collection and treatment, data analysis of mass-spectrum, draft of the manuscript. BBZ: data analysis of mass-spectrum, revise the article. ML: direct and help to the experiment. KFD: serum collection and treatment. MH: direct and help to the experiment. All authors read and approved the final manuscript.
